# Analytic Review of Modeling Studies of ARV Based PrEP Interventions Reveals Strong Influence of Drug-Resistance Assumptions on the Population-Level Effectiveness

**DOI:** 10.1371/journal.pone.0080927

**Published:** 2013-11-25

**Authors:** Dobromir Dimitrov, Marie-Claude Boily, Elizabeth R. Brown, Timothy B. Hallett

**Affiliations:** 1 Vaccine & Infectious Disease Division, Fred Hutchinson Cancer Research Center, Seattle, Washington, United States of America; 2 Department of Infectious Disease Epidemiology, Faculty of Medicine, Imperial College London, London, United Kingdom; 3 Department of Applied Mathematics, University of Washington, Seattle, Washington, United States of America; 4 Department of Biostatistics, University of Washington, Seattle, Washington, United States of America; Imperial College London, United Kingdom

## Abstract

**Background:**

Four clinical trials have shown that oral and topical pre-exposure prophylaxis (PrEP) based on tenofovir may be effective in preventing HIV transmission. The expected reduction in HIV transmission and the projected prevalence of drug resistance due to PrEP use vary significantly across modeling studies as a result of the broad spectrum of assumptions employed. Our goal is to quantify the influence of drug resistance assumptions on the predicted population-level impact of PrEP.

**Methods:**

All modeling studies which evaluate the impact of oral or topical PrEP are reviewed and key assumptions regarding mechanisms of generation and spread of drug-resistant HIV are identified. A dynamic model of the HIV epidemic is developed to assess and compare the impact of oral PrEP using resistance assumptions extracted from published studies. The benefits and risks associated with ten years of PrEP use are evaluated under identical epidemic, behavioral and intervention conditions in terms of cumulative fractions of new HIV infections prevented, resistance prevalence among those infected with HIV, and fractions of infections in which resistance is transmitted.

**Results:**

Published models demonstrate enormous variability in resistance-generating assumptions and uncertainty in parameter values. Depending on which resistance parameterization is used, a resistance prevalence between 2% and 44% may be expected if 50% efficacious oral PrEP is used consistently by 50% of the population over ten years. We estimated that resistance may be responsible for up to a 10% reduction or up to a 30% contribution to the fraction of prevented infections predicted in different studies.

**Conclusions:**

Resistance assumptions used in published studies have a strong influence on the projected impact of PrEP. Modelers and virologists should collaborate toward clarifying the set of resistance assumptions biologically relevant to the PrEP products which are already in use or soon to be added to the arsenal against HIV.

## Introduction

Several randomized clinical trials demonstrated that if used adequately, pre-exposure prophylaxis (PrEP) products in the form of daily pills (oral PrEP) or topical gels (vaginal microbicides) could be partially effective in preventing HIV acquisition for men who have sex with men [1], heterosexual men and women [2], [3], and serodiscordent couples [4]. Although these encouraging results were not confirmed in two other trials [5], [6], Truvada (emtricitabine/tenofovir disoproxil fumarate) became the first drug approved for PrEP use in US [7]. Guidelines for the safe use of PrEP by MSM and heterosexually active adults have recently been published by the Center for Diseases Control (CDC) and the Southern African HIV Clinicians Society [8]–[10]. PrEP products could therefore add to the HIV prevention options available to both state and local health officials, who then decide how best to allocate resources.

Mathematical models are increasingly being used to assess the potential public health impact of oral or topical PrEP interventions for specific regions or populations in order to better understand when they can be most useful [11]–[30]. One of the greatest concerns for the successful addition of antiretroviral (ARV) based PrEP to HIV prevention programs is that infected individuals who use PrEP, because they are either unaware of their serostatus or become HIV-positive after starting to use the product, may acquire drug resistance (ADR) and thus increase the risk of transmitting drug resistance (TDR). Currently, little resistance was reported in the completed clinical trials, which may be at least partly because the trials were of short duration and because PrEP users were frequently monitored and taken off treatment soon after seroconversion, probably much sooner than would likely happen in real life.

Of all modeling studies assessing the effectiveness of PrEP published before 2012 [11]–[30], only a few discussed the possible spread of drug resistance and its influence on the success of PrEP interventions. These mathematical models use a broad range of assumptions and parameter values to reflect the mechanisms of drug-resistance (e.g. the rate of emergence of drug resistance among HIV positive individuals who use PrEP, infectiousness of resistance carriers, rate of resistance reversion following PrEP discontinuation, and PrEP efficacy against drug-resistant HIV infection). In addition, modeling studies examine various PrEP intervention scenarios in different HIV epidemics and populations, often using study-specific indicators and time frames to assess the benefits and risks associated with PrEP use. Therefore, differences in the projected population-level impact of PrEP use across models are difficult to understand, even in in-depth reviews [31]. Depending on the combination of assumptions used, some modeling studies suggest that the emergence of drug resistance may lead to a greater reduction in overall HIV incidence.

The HIV Modeling Consortium, which aims to help improve scientific support for decision making by co-coordinating a wide range of research activities in mathematically modeling the HIV epidemic, has acknowledged the importance of the issues related to PrEP resistance. At a meeting organized by the consortium in 2011, mathematical modelers and virologists discussed model and parameters assumptions required to represent the potential emergence and spread of drug resistance due to PrEP. Participants agreed that resistance mechanisms embedded in models should be rigorously and systematically studied [32].

In this paper, we extend the narrative review by Baggaley et al [31] and investigate, with a mathematical model, the independent and combined effects of key resistance assumptions from previously published studies on the outcomes of oral PrEP interventions. We focus on understanding the extent to which ignoring the development and spread of resistance influences modeling results, under a variety of epidemic conditions. We also identify conditions under which drug resistance may cause an increase in the projected number of prevented infections due to PrEP use.

## Methods

### Transmission model

**Figure 1:**
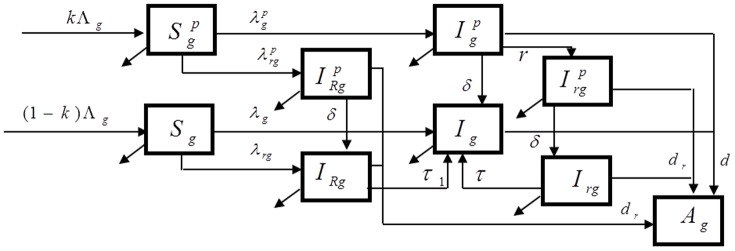
Flow diagram of the model. Simulated population is stratified in compartments by gender as men (subscript g = m) or women (subscript g = w) and by HIV status as susceptibles (S), infected with wild HIV (I), infected with drug-resistant HIV through transmission (I_R_), individuals who developed (acquired) resistance on PrEP (I_r_) and AIDS (A). PrEP users (superscript p) strictly follow the prescribed daily regimen but drop out (at rate δ) if tested HIV positive. A complete description of the model including the expressions for the forces of infections (λ) is presented in File S1.

We developed a compartmental mathematical model of HIV transmission in a heterosexual population (age 15–49) to study the influence of drug resistance on the impact of PrEP (Figure 1). The individuals in the simulated population are aggregated in compartments for men (subscript g = m) and women (subscript g = w), by PrEP status as users (superscript p) and nonusers, and by HIV status as susceptible (S), infected with wild-type HIV (I), infected with drug-resistant HIV through transmission (subscript R), individuals who developed (acquired) resistance while using PrEP (subscript r) and AIDS (A). HIV susceptible men and women who become sexually active join the community at constant rates, and are selected to balance the departure rate in an uninfected population. The rates at which men and women acquire HIV-infection, i.e., forces of infection for different classes, are derived from standard binomial models based on the annual number of partners per susceptible person, the number of sex acts per partnership, the fraction of sex acts protected by condoms, and the HIV acquisition risk per vaginal act for men and women, which may depend on resistance status of the infected partner at the time of exposure (wild type vs. drug-resistant carrier). A fraction of the initial and newly recruited population is assumed to be PrEP users. When PrEP is used by one or both partners in a serodiscordant contact, the acquisition risk of the susceptible partner is influenced by the protection per sex act provided by PrEP (PrEP efficacy) as described below. PrEP users may undergo initial and subsequent periodic HIV testing at different frequencies. They strictly follow the prescribed daily regimens but are removed from PrEP if tested positive for HIV. The efficacy of PrEP is allowed to vary for resistant infections as described in one of the next sections. A complete description of the model is given in File S1.

### PrEP efficacy and intervention coverage

The results from the concluded clinical trials suggest that when used consistently, oral PrEP provides partial protection against HIV acquisition and therefore reduces the susceptibility of the users [1], [3], [4]. It is also theoretically plausible that infected individuals who continue to use ARV-based PrEP will have a reduced viral load and therefore a lower risk of transmitting HIV to their uninfected partners. Studies of tenofovir use by HIV-positive individuals have associated it with low-level viremia [33]–[35], and support the potential role of PrEP in reducing infectiousness incorporated in some of the published modeling studies. Here we assume equal reduction of susceptibility and infectiousness due to PrEP use. The protective effect is multiplicative when both partners in a serodiscordant contact use PrEP. To ensure comparability between all scenarios we assume that 50% of the sexually active men and women start using PrEP from the moment of its introduction (instantaneous uptake) and follow strictly the prescribed regimen (perfect adherence). The same fraction (50%) of newly sexually active individuals is assumed to become PrEP users. Although important when predicting the absolute effectiveness of PrEP interventions, coverage is unlikely to affect the relative contribution of resistance, which we investigate (see Fig. S1 in File S1 for simulations with 25% coverage). Efforts to limit PrEP use by HIV positive individuals are incorporated in the intervention at introduction and are followed by periodic HIV screening. No change in sexual behavior (frequency of sex acts, condom use rate) due to the access to PrEP is considered. A complete description of the intervention parameters, unrelated to resistance, is given in Table 1, part C.

**Table 1:** Parameters and ranges used in the analysis.

Parameter	Description	Values and ranges	Ref.
**A. Behavioral and epidemic parameters (pre-intervention)**		**Prior range**	
β_w_	Female HIV acquisition risk per vaginal act	0.0019–0.0046	[38]
β_m_	Male HIV acquisition risk per vaginal act	(50–100%) of β_w_	assumed [38]
1/μ	Average time to remain sexually active	35 years	[39], [40]
d_r_, d	HIV-related mortality rates of people infected with resistant and wild-type HIV	8.3%–14.3%	[41], [42]
n_w_, n_m_	Average number of sexual acts per year for women and men	60–100	[43], [44]
ρ_w_, ρ_m_	Average number of sexual partners per year for women and men	0.5–1.5	[44]–[45]
c	Rate of condom use in general population as a fraction of sex acts in which a condom is used	20–60%	[43], [44]
α_c_	Condom efficacy per sex act	0.8–0.95	[46]
**B. Calibrated epidemic data**			
Pw	Initial HIV-prevalence (women)	20%	[36]
Pm	Initial HIV-prevalence (men)	15%	[36]
Inc	Fitted HIV-incidence (total)	0.6–2.5%	[37]
Pr_5_	Fitted HIV-prevalence in 5 years (total)	16.5%–18.5%	assumed
**C. Intervention parameters**		**Baseline value**	
k	PrEP coverage. Proportion of men and women who use PrEP.	50%	assumed
k_1_	Initial fraction of the susceptibles using PrEP	50%	assumed
α_S_	PrEP efficacy in reducing susceptibility per act when exposed to wild-type HIV	50%	assumed
α_i_	PrEP efficacy in reducing infectiousness per act wild type when exposed to wild-type HIV	50%	assumed
γ	PrEP adherence	100%	assumed

### Assumption on drug resistance due to ARV PrEP

Consistent PrEP use after HIV acquisition or initiating PrEP in already infected individuals may lead to emergence of acquired drug resistance (ADR), which may subsequently be transmitted to ARV-naïve individuals (transmitted drug-resistance, TDR). We reviewed 20 studies, published before 2012, which evaluated the impact of oral or topical PrEP with dynamic mathematical models [11]–[30], and focused on the studies which addressed drug resistance due to PrEP [15], [19], [21], [23]–[26]. Three of the selected studies simulate interventions of topical PrEP [19], [23], [25] while the other four investigate interventions of oral PrEP [15], [21], [24], [26]. We extracted the modeling and parameter assumptions regarding the emergence, persistence and transmission of drug resistance due to PrEP implemented in these models. Our model, which represents oral PrEP, was designed to accommodate key resistance-related mechanisms and assumptions used in the previous published analyses in order to compare their influence. The list includes assumptions regarding the efforts to restrict the access of infected individuals to PrEP, which could be managed during real-time interventions by:

Restricting the opportunities of already infected individuals to start using PrEP. Prescription rejection rate (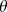) represents the actual reduction in new PrEP prescriptions issued to infected compared to susceptible individuals. If the access to PrEP is not controlled (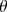 = 0), then the same proportion of infected and susceptible people initiate PrEP. Conversely, if the access to PrEP is under strict control (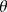 = 1), then no infected individuals start using PrEP;
Removal of the HIV-positive PrEP users from PrEP as a result of periodic HIV screening. Annual PrEP drop-rate (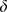) represents the rate at which infected PrEP users stop taking their pills. It is reciprocal to the average time to remain on PrEP after HIV acquisition. For instance, if 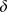 = 0.5 people who acquire HIV when on PrEP continue to take their pills for an average of two years.

The rest of the modeling assumptions are related to the natural history of resistance in an infected host using PrEP:

Consistent use of oral PrEP by infected individuals leads to emergence of drug resistance at rate 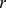. The resistance rate is reciprocal to the average time needed to develop resistance while infected with HIV and using PrEP;
Resistance carriers may be less infectious than those infected with wild-type HIV (relative infectiousness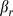) due to the reduced fitness of the resistant compared to wild-type HIV. Relative infectiousness of 50% (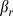 = 0.5) implies that it is half as likely to acquire HIV during contact with a partner infected with resistant HIV;
Probabilities to acquire resistant HIV through transmission, given that the transmission occurs, may be different for contacts with partners infected with TDR (probability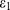) and ADR (probability 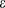);
The dominance of resistant HIV in an infected host may revert back to wild type when PrEP is not used. The reversion rate may be substantially slower in ARV-naïve hosts with TDR (reversion rate 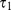) compared to former PrEP users who developed ADR when on PrEP (reversion rate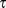). For instance, ADR may revert back over three months after PrEP use is interrupted (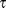 = 4), while it may take four years to lose detectable TDR (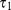 = 0.25);
PrEP may provide reduced protection when susceptible users are exposed to drug-resistant compared to wild-type HIV (relative PrEP efficacy 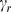). A relative PrEP efficacy of 50% (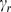 = 0.5) implies that PrEP is half as effective per sex act with a partner infected with resistant HIV compared to an act with a partner infected with wild-type HIV.

We have compiled a list of the ranges of resistance-related parameters used in the published papers which include resistance (see Table 2). To help interpret results across studies, we analyzed the model outcomes using 10,000 parameter combinations, randomly sampled from the set extracted from each study. An aggregated parameter set (Table 2, last column), which combines the ranges from all studies, is used when the impact of resistance factors is evaluated in multivariate sensitivity analysis.

**Table 2:** Resistance-related parameters used in the analysis.

Description (symbol)	Parameters sets extracted from published papers							Baseline value
	P 1 [19]	P 2 [23]	P 3 [25]	P 4 [26]	P 5 [21]	P 6 [15]	P 7 [24]	(Range)^2^
Rate of resistance development by full adherers to PrEP (r)	0–2	0–2	0.5–1	2.6–27.6	0.1–1	0.5–1	2–6	3 (1–5)
Prescription rejection rate (θ) which measures the reduction in the initial fraction of HIV-positive individuals who initiate PrEP (1-θ)k_1_ compared to the corresponding fraction of HIV-negative individuals k_1_	0	0–1	0.1–0.7	1	1	1	0.75–0.95	75% (50%–100%)
Annual PrEP dropout rate by HIV-positive individuals (δ)	0	0–1	0–1	2–4	2–333	0.33–2^1^	0.33–2	1 (0.33–3)
Rate of resistance reversion for HIV-positive former PrEP users who acquired drug resistance when on PrEP (τ)	0	0	0–4	1–4	2–12	1–12	1–12	4 (2–6)
Rate of resistance reversion for HIV-positive non-users of PrEP to whom the resistant HIV has been transmitted (τ_1_)	0	0	0–4	0.07–0.5	0.07–0.5	2–12	0.2–1	0.3 (0.1–0.5)
Probability to transmit resistant over wild-type HIV by infected individuals with ADR (ε)	1	1	1	1	1	0.2–1	0.5–1	75% (50–100%)
Probability to transmit resistant over wild-type HIV by infected individuals with TDR (ε_1_)	1	1	1	1	1	0.5–1	0.75–1	87.5% (75–100%)
Relative infectiousness of individuals infected with resistant HIV (β_r_)	0.05–0.5	0.05–0.5	0.25	0.01–0.6	0.75–1	0.5–1	0.5–1	50% (0–100%)
Relative PrEP efficacy when exposed to resistant compared to wild type HIV (γ_r_)	1	1	1	0–0.5	0–0.5	0–0.5	0–0.25	50% (0–100%)

1 No values are provided in the paper. The range from the more recent paper [24] of the same research group is used.

2 Theranges for the resistance-related parameters are used in the multivariate sensitivity analysis (see File S1).

### Epidemic settings and intervention scenarios

Demographic, behavioral and epidemic parameters were defined and initially sampled from ranges representative of the HIV epidemics in the Sub-Saharan region (see Table 1, part A). Next, we identified 1,000 posterior parameter sets, reflecting prototypical hyper endemic in southern Africa using the following target criteria (see Table 1, part B): i) initial HIV prevalence of 15% and 20% among 15–49 years old men and women, respectively [36]; ii) annual incidence rate between 0.6% and 2.5% [37]; iii) female incidence rate at least 30% higher than male incidence rate[37]; and iv) the absolute difference in HIV prevalence over five years remains below 1% (mature epidemics).

With the selected epidemic parameters we simulate: i) an HIV epidemic without PrEP use; ii) an HIV epidemic with PrEP use but without drug resistance due to PrEP, and iii) an HIV epidemic with PrEP use assuming emergence of drug resistance due to PrEP. The impact of the PrEP interventions is measured in terms of the cumulative fraction of infections prevented (CPF), the cumulative fraction of infections in which drug-resistant HIV is transmitted (TRF), and the resistance prevalence (RP) among HIV-positive individuals, all evaluated over ten years of PrEP use. The choice of epidemic sets has little influence on the intervention outcomes. Results from simulations in which only epidemic and resistance parameters are varied while the other parameters remain fixed are presented in the Supporting Information (see Fig. S2 in File S1). To delineate the influence of the resistance on the projected PrEP effectiveness we evaluate the relative CPF, defined as the ratios of CPF for scenarios with resistance over scenarios without resistance: 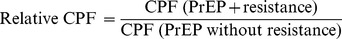



Values of the relative CPF greater than one indicate that the addition of resistance improves the CPF. The contribution of the resistance to the PrEP effectiveness is measured by the relative change in the projected CPF under scenarios with and without resistance:
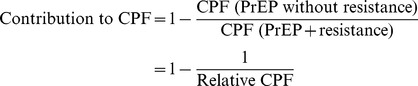



This metric estimates what fraction of the CPF can be attributed to the resistance. The contribution of the resistance is positive (i.e. reduce HIV incidence) when the relative CPF is greater than one.

The influence of resistance parameters on key intervention outcomes (relative CPF and resistance prevalence after ten years of PrEP use) is studied in a multivariate sensitivity analysis. Partial rank correlation coefficients (PRCC) are calculated for each parameter-outcome pair to evaluate the impact of parameter variation on the monotonicity of the outcome. The analysis is based on 10,000 simulations using randomly sampled resistance parameters and intervention parameters fixed on their baseline values from Table 1, part C. Additional sensitivity analysis results are presented in the Supporting Information (see Fig. S3 in File S1).

## Results

### Summary of resistance assumptions from the review of published PrEP models

The resistance parameters used in the published modeling studies demonstrate enormous variability in assumptions and uncertainty in values (see Table 2). All the papers (except [19]) include activities to reduce the PrEP use by infected individuals (positive prescription rejection rate θ and drop rate δ). However, some studies assume “susceptible only” access to PrEP (θ = 1) [15], [21], [26], while others [23], [25] explore wide ranges corresponding to interventions with unrestricted access (θ = 0) to “susceptible only” access (θ = 1). The rates at which infected PrEP users acquire resistance (r) also vary substantially across studies. The range explored in [19], [23] suggests that it takes from six months to indefinitely longer for resistance to develop, while the same time is estimated at between one and six months in [24], [26]. The majority of the studies (all except [19] and [23]) allow for the resistant HIV to revert back to wild-type HIV when PrEP is not used, with four of the models differentiating the reversion rates for acquired and transmitted resistance [15], [21], [24], [26]. Five of the models assume that the carriers of resistance transmit only resistant HIV [19], [21], [23], [25], [26], while the remaining two allow also for wild-type HIV to be transmitted with probability of up to 50% and 80%, respectively. All published models agree that the infectiousness of the resistance carriers will be decreased due to fitness loss of the resistant HIV, but the reduced levels explored in each paper are fundamentally different. Only four of the studies consider the possibility that PrEP will provide limited protection against resistant HIV [15], [21], [24], [26].

### Contribution of drug resistance to the population-level PrEP impact

**Figure 2:**
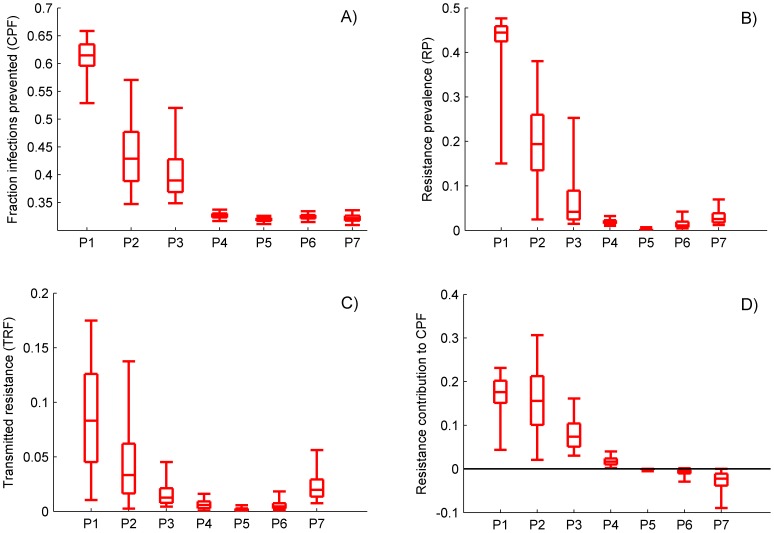
Public-health impact of 10 years of consistent PrEP use by 50% of the population projected by the model parameterized with the assumptions extracted from published papers. The outcomes presented are A) the cumulative fraction of prevented infections (CPF); B) resistance prevalence due to PrEP (RP); C) cumulative fraction of infections in which resistance is transmitted (TRF) and D) resistance contribution to CPF. The boxplots (median, 2.5th, 25th, 75th, 97.5th percentiles) reflect the variation in impact estimates based on 10,000 simulations (10 per preselected epidemic set). In D, the contribution of resistance to CPF is calculated as the percentage change in CPF from simulations in which the resistance is disregarded. Intervention parameters are fixed on their baseline values from Table 1, part C.

Figure 2 shows the impact of resistance on the predicted PrEP benefits (ten-year CPF) and PrEP risks (RP and TRF) using resistance parameters from different modeling studies. The influence of resistance on the number of infections prevented varied considerably across studies despite the same behavioral, epidemic and intervention conditions. The projected ten-year CPF varied between 32% and 62% (Fig. 2A), the predicted resistance prevalence among infected individuals varied between from 2% to 44% (Fig. 2B), and the infections in which resistance is transmitted varied between 1% and 8% (Fig. 2C). The two sets (P1 and P2) that produce the most optimistic predictions in terms of CPF (43%–62%) also predict the largest resistance prevalence (20%–44%). If the model is parameterized with the set P1 the resistance has a substantial contribution (a median of 18%) to the PrEP effectiveness, which translates into up to 11% of the total HIV infections additionally prevented due to resistance over ten years (Fig. 2D). In contrast, the model using parameter sets P4–P7 projects only 32%–33% infections prevented over ten years, with a minimal variation due to the resistance assumptions. Note that the resistance prevalence due to PrEP with the parameter set P5 remains below 1% in 97.5% of the simulated epidemics, while the results obtained with set P7 predict approximately the same reduction in HIV infections over ten years, but a larger proportion of resistance among HIV-positive individuals (up to 7% in some simulations). Under the parameterization with P4 resistance still adds to CPF (its contribution is up to 5%), while the simulations using P7 show negative contribution of resistance to CPF, i.e., actual reduction of CPF by up to 10%.

The magnitude of the resistance contribution (positive or negative) is limited when stronger restrictions on PrEP use by HIV positive individuals are imposed. All sets except P1 incorporate restricted access to PrEP (positive prescription rejection rates θ and PrEP drop rates δ) for infected individuals, but the stronger control assumed in the sets P4–P7 (θ near 1 and large δ) leads to a substantially smaller number of infected people who use PrEP, i.e, being at risk of emerging resistance. Another factor limiting resistance effects is the reversion of the resistant HIV back to wild-type after the interruption of PrEP use. It is included in all sets except P1 and P2 (positive τ and τ_1_). The faster reversion assumed in sets P4–P7 implies short persistence of resistance when PrEP is not used, and smaller influence of the resistance on the intervention outcomes.

The large uncertainty in the projections with sets P1 and P2 is driven by the wide range for the average time (1/r) needed to acquire resistance when infected and using PrEP (between six months and never), unrestricted or weakly-restricted access to PrEP by infected individuals, and irreversible resistance status after PrEP is interrupted. This allows for accumulation of a substantial number of infected individuals using PrEP who in some scenarios quickly acquire persistent resistance, while in others may never develop ADR. Conversely, when the infected users are quickly removed from PrEP and the reversal of drug resistance back to wild-type does not take long, the influence of resistance on the intervention outcomes remains low (sets P4 and P5) under all epidemic conditions.

### When is drug resistance beneficial?

The projections of our model with different parameter sets suggest that the possibility for the resistance to contribute positively to PrEP effectiveness (Fig.2D, P1–4) is utilized by two modeling assumptions: i) the influence of the reduced viral fitness of the drug-resistant HIV on its ability to be effectively transmitted and ii) the loss of PrEP protection due to an exposure to drug-resistant compared to wild-type HIV, which act in opposite directions. The loss of viral fitness lowers the infectiousness of the carriers of resistance (*relative infectiousness* 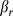) and indirectly increases the effectiveness of PrEP, while the reduced PrEP efficacy against resistant HIV (*relative* PrEP *efficacy* 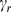) decreases the number of infections prevented due to PrEP, and as a result decreases the effectiveness of the interventions. Parameter sets, which assume that the PrEP efficacy is the same against resistant and non-resistant HIV (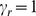) and that resistance carriers are significantly less infectious (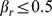), predict positive contribution of the resistance to CPF (Fig. 2D, P1–P3). A negative resistance contribution to CPF (Fig. 2D, P5–P7) is observed when a relatively low PrEP efficacy against resistant HIV (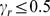) is combined with a small fitness cost of resistance (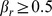).

**Figure 3:**
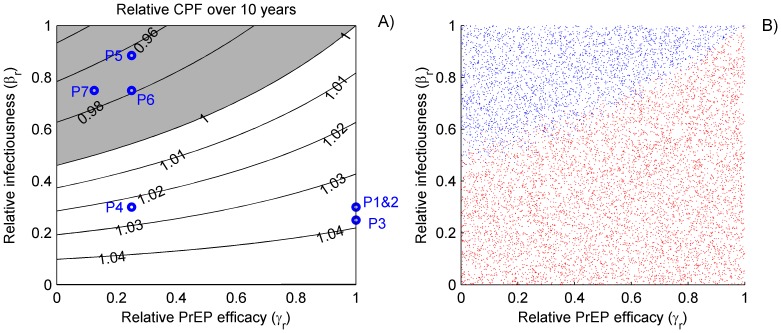
Impact of the relative PrEP efficacy (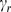, 0 – no protection, 1 – equal protection) when exposed to drug-resistant compared to wild type HIV and the relative infectiousness (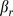, 0 – not infectious, 1 – equally infectious) of individuals infected with drug-resistant compared to wild-type HIV on the resistance contribution to the cumulative fraction of infections prevented (CPF) over 10 years. A) Contour plots of the relative CPF with all resistance parameters, except 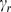 and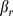, fixed on their baseline values from Table 2 (last column). Values less than one (shaded region) indicate that resistance impacts negatively the estimate of the CPF. Blue dots represent the median value of 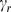 and 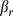
from the parameter sets defined in Table 2; and B) Simulations with positive (red) and negative (blue) resistance contributions using resistance parameters randomly sampled from the ranges in Table 2 (last column).; Relative CPF is calculated as the ratio of the 10-year CPF for scenarios with resistance over baseline scenario (no resistance).

We study further the impact the *relative infectiousness* (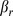) and *relative* PrEP *efficacy* (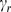) on the relative CPF with the rest of the resistant parameters fixed (Fig.3A) or varied in their ranges from Table 2 (Fig.3B). This analysis confirms that the resistance is more likely to be beneficial in terms of CPF if the infectiousness of drug resistance carriers is substantially reduced (*relative infectiousness* below 50%) compared to the wild-type HIV carriers. If PrEP provides the same protection against resistant and wild-type HIV (*relative efficacy* equals one), then resistance always improves the CPF independently of *relative infectiousness* of the infected with resistant HIV. Overall, resistance has negative effects on CPF only if PrEP is substantially less effective against resistant HIV, and resistance carriers are almost as infectious as wild-type carriers (shaded region in Fig. 3A and blue region in Fig. 3B). The perfect alignment between these regions and the clear separation between blue and red plots in Fig. 3B suggest that the impact of the remaining resistance parameters on the relative CPF is negligible. Therefore, the combination of *relative infectiousness* and *relative efficacy* used in a mathematical model could be a good predictor of whether the inclusion of resistance increases or reduces the projected PrEP effectiveness. The median values of the *relative infectiousness and efficacy* from the published studies plotted in Fig.3A are sufficient to correctly predict the direction of resistance contribution (positive for P1–P4 and negative for P5–P7) when corresponding parameter sets are used (Fig. 2D).

### Sensitivity analysis

**Figure 4:**
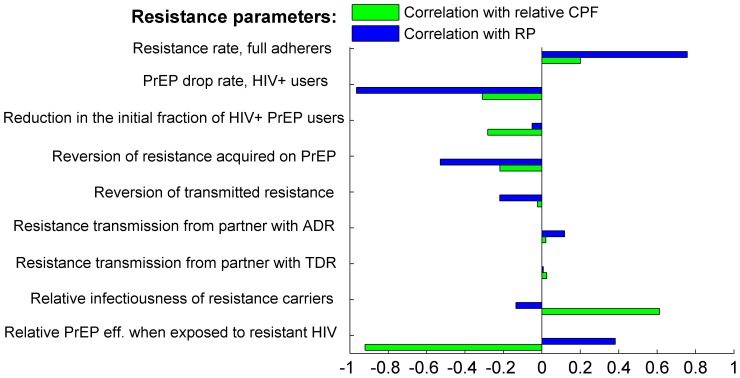
Partial rank correlation coefficients (PRCC) between resistance-related parameters and intervention outcomes, relative 10-year CPF (green) and resistance prevalence after 10 years (blue) based on 10, 000 simulations (10 per preselected epidemic set). The intervention parameters are fixed on their baseline values from Table 1, part C. Relative CPF is calculated as the ratio of the 10-year CPF for scenarios with resistance over baseline scenario (no resistance).

The partial rank correlation coefficients (PRCC) between resistance parameters and intervention outcomes (relative CPF and resistance prevalence after ten years) are presented in Figure 4. It clearly confirms the observation from the previous section that only the relative PrEP efficacy against resistant HIV and relative infectiousness of the resistance carriers have a strong impact on the monotonicity of the relative ten-year CPF. The restriction of the PrEP use by infected individuals and the resistance reversion rate are among the key factors, negatively correlated with the resistance prevalence. Not surprisingly, the rate to develop resistance while infected and using PrEP is the strongest driver of the expected resistance prevalence and therefore needs to be carefully monitored when a particular PrEP intervention is rolled out.

## Discussion

Possible spread of drug-resistance due to the availability of PrEP is a major public health concern when deciding to include PrEP in HIV prevention programs. In this study we simulated and objectively compared the influence of different resistance assumptions on the predicted population-level impact of PrEP. We have borrowed PrEP resistance profiles from previously published modeling studies and tested the extracted parameter sets under identical epidemic conditions and intervention scenarios. This analytic approach allowed us to assess the impact of each resistance assumption on intervention outcomes in order to guide the interpretation of these in further studies. We found that a wide range of resistance prevalence (from 1% to almost 50%) may be expected after ten years of PrEP use if 50% effective PrEP is consistently used by 50% of the population (Fig. 2B). The spread of resistance may contribute both negatively (up to 10%) or positively (up to 30%) to the projected PrEP effectiveness, depending on what resistance parameterization has been used (Fig. 2D).

From a public-health perspective our analysis implies that the influence of resistance should not be neglected. The large impact projected under scenarios with weak control on the access to PrEP by infected individuals and slow reversion back to wild-type after PrEP is interrupted suggests that PrEP users have to be carefully monitored in future community interventions. Modeling studies which do not address the emergence of resistance not only underestimate the risk, but may predict PrEP impact at significantly different levels compared to analyses in which resistance is included (up to 30% under some of the extracted sets of resistance assumptions). Although we are not in a position to recommend or reject any specific resistance-handling setups, we would like to point out the critical importance of the way resistance is incorporated in the modeling studies.

Many different factors should be considered when the mechanisms of emergence and persistence of drug-resistance due to PrEP are analyzed. We identified two of them: the decreased PrEP efficacy against resistant HIV, and the reduction in HIV fitness (decreased infectiousness of resistance carriers), which in combination may serve as a predictor if whether drug resistance will add to the projected PrEP effectiveness (Fig. 3). Although under no conditions should the spread of resistant HIV be considered a positive outcome, this result is informative, for the efforts to resolve the problems with resistance may improve or reduce the overall impact of the intervention. More empirical data is needed to obtain better estimates of those parameters for the promising PrEP candidates.

Several simplifying assumptions, made in the model, may influence the presented results. For instance, the contribution of resistance is amplified by the reduced infectiousness of PrEP users applied in the model. This assumption, incorporated in four of the published studies [19], [21], [23], [25], is based on the expected lower viral load of individuals who become infected while using PrEP (breakthrough infections). Modeling the adherence to PrEP present additional difficulties because the effects of the inconsistent PrEP use on the PrEP efficacy and the rate of resistance emergence is unclear. Our simplifying assumption of perfect adherence avoids these uncertainties but may overestimate the projected impact of PrEP. However, it does not affect the comparison of the extracted sets of resistance assumptions since we use the resistance rates from the published papers which correspond to perfect adherence. The epidemic conditions, intervention assumptions, the structure and complexity of the model, and the sampling methods of the input parameters used in our analysis differ from the original studies. Therefore, the results reported are not comparable to those reported in the previously published papers and are representative of the extracted sets of resistance assumptions only.

This analysis presents a novel approach of identifying and investigating the importance of specific mechanisms and assumptions to the impact evaluations based on mathematical models. Our findings invite extensive communication between modelers and virologists toward clarifying the set of resistance assumptions biologically relevant to the PrEP products which are already in use or may be soon added to the arsenal against HIV. We should reiterate that our projections are related to resistance due to PrEP only and do not track resistance from other sources, such as failing ART. It is possible that the carriers of PrEP resistance may have increased chance to fail ART when initiated if PrEP and ART used at the same location share active components. Therefore, the increasing number of people using PrEP should be closely monitored and all breakthrough infections should be tested for drug-resistance. Data on the rates of resistance development, reversion of resistance, fitness loss of the drug-resistant HIV and rates of failing ART among those who become infected while using PrEP is critically needed to improve the accuracy of the resistance projections obtained through mathematical models.

## Supporting Information

File S1 Supporting Information
(DOCX)

## References

[1] Grant RM, Lama JR, Anderson PL, McMahan V, Liu AY, et al (2010) Preexposure Chemoprophylaxis for HIV Prevention in Men Who Have Sex with Men. New England Journal of Medicine 363: 2587–2599. 21091279 10.1056/NEJMoa1011205 PMC3079639

[2] Abdool Karim Q, Abdool Karim S, Frohlich J, Grobler A, Baxter C, et al (2010) Effectiveness and safety of tenofovir gel, an antiretroviral microbicide, for the prevention of HIV infection in women, Science. 329(5996): 1168–1174. 10.1126/science.1193748 PMC3001187 20643915

[3] Centers of Disease Control and Prevention (2011) CDC Trial and Another Major Study Find PrEP Can Reduce Risk of HIV Infection among Heterosexuals, Press Release. Available: http://www.cdc.gov/nchhstp/newsroom/PrEPHeterosexuals.html. Accessed 04 January 2013.

[4] Baeten JM, Donnell D, Ndase P, Mugo NR, Campbell JD, et al (2012) Antiretroviral Prophylaxis for HIV Prevention in Heterosexual Men and Women. New England Journal of Medicine 367: 399–410. 22784037 10.1056/NEJMoa1108524 PMC3770474

[5] FHI (2011) FHI Statement on the FEM-PrEP HIV Prevention Study, FHI360, Press Release. Available: http://www.fhi360.org/en/AboutFHI/Media/Releases/FEM- PrEPstatement041811.htm. Accessed 04 January 2013.

[6] Jeanne Marrazzo GR, Nair G, Palanee T, Mkhize B, Nakabiito C, et al.. (2013) Pre-exposure Prophylaxis for HIV in Women: Daily Oral Tenofovir, Oral Tenofovir/Emtricitabine, or Vaginal Tenofovir Gel in the VOICE Study (MTN 003). Conference on Retroviruses and Opportunistic Infections. Atlanta, GA.

[7] Food and Drug Administration (2012) FDA approves first drug for reducing the risk of sexually acquired HIV infection. Press Release. Available: http://www.fda.gov/NewsEvents/Newsroom/PressAnnouncements/ucm312210.htm. Accessed 04 January 2013.

[8] Centers of Disease Control and Prevention (2011) Interim Guidance: Preexposure Prophylaxis for the Prevention of HIV Infection in Men Who Have Sex with Men. Morbidity and Mortality Weekly Report 60(03): 65–68. 21270743

[9] Centers of Disease Control and Prevention (2012) CDC Issues Interim Guidance on Use of Medication to Prevent HIV Infection among Heterosexually Active Adults, Press Release. Available: http://www.cdc.gov/nchhstp/newsroom/2012/PrEP-HeterosexualGuidance-PressRelease.html. Accessed 04 January 2013.

[10] The Consensus Committee, Southern African HIV Clinicians Society, chaired by Linda-Gail Bekker and Kevin Rebe (2012) Southern African guidelines for the safe use of pre-exposure prophylaxis in men who have sex with men who are at risk for HIV infection, Southern African Journal of HIV Medicine 13. (2): 40–55.

[11] Karmon E, Potts M, Getz WM (2003) Microbicides and HIV: Help or hindrance? JAIDS 34: 71–75. 14501797 10.1097/00126334-200309010-00011

[12] Breban R, McGowan I, Topaz C, Schwartz EJ, Anton P, et al (2006) Modeling the potential impact of rectal microbicides to reduce HIV transmission in bathhouses. Mathematical Biosciences and Engineering 3: 459–466. 20210374 10.3934/mbe.2006.3.459

[13] Chen FH (2006) The impact of microbicides and changes in condom usage on HIV prevalence in men and women. AIDS 20: 1551–1553. 16847411 10.1097/01.aids.0000237372.38939.5d

[14] Vickerman P, Watts C, Delany S, Alary M, Rees H, et al (2006) The importance of context: Model projections on how microbicide impact could be affected by the underlying epidemiologic and behavioral situation in 2 African settings. Sexually Transmitted Diseases 33: 397–405. 16721331 10.1097/01.olq.0000218974.77208.cc

[15] Abbas UL, Anderson RM, Mellors JW (2007) Potential Impact of Antiretroviral Chemoprophylaxis on HIV-1 Transmission in Resource-Limited Settings. PLoS ONE 2: e875. 17878928 10.1371/journal.pone.0000875 PMC1975470

[16] Desai K, Sansom SL, Ackers ML, Stewart SR, Hall HI, et al (2008) Modeling the impact of HIV chemoprophylaxis strategies among men who have sex with men in the United States: HIV infections prevented and cost-effectiveness. AIDS 22: 1829–1839. 18753932 10.1097/QAD.0b013e32830e00f5

[17] Vissers DCJ, Voeten HACM, Nagelkerke NJD, Habbema JDF, de Vlas SJ (2008) The Impact of Pre-Exposure Prophylaxis (PrEP) on HIV Epidemics in Africa and India: A simulation Study. PLoS ONE 3: e2077. 18461185 10.1371/journal.pone.0002077 PMC2367053

[18] Vickerman PD, Foss AP, Watts CP (2008) Using Modeling to Explore the Degree to Which a Microbicide's Sexually Transmitted Infection Efficacy May Contribute to the HIV Effectiveness Measured in Phase 3 Microbicide Trials. JAIDS 48(4): 460–467. 18614928 10.1097/QAI.0b013e31817aebd6

[19] Wilson DP, Coplan PM, Wainberg MA, Blower SM (2008) The paradoxical effects of using antiretroviral-based microbicides to control HIV epidemics, Proceedings of The National Academy of Sciences of The United States of America. 105: 9835–984. 10.1073/pnas.0711813105 PMC2447864 18606986

[20] Paltiel AD, Freedberg KA, Scott CA, Schackman BR, Losina E, et al (2009) HIV Preexposure Prophylaxis in the United States: Impact on Lifetime Infection Risk, Clinical Outcomes, and Cost-Effectiveness. Clinical Infectious Diseases 48: 806–815. 19193111 10.1086/597095 PMC2876329

[21] Supervie V, Garcia-Lerma JG, Heneine W, Blower S (2010) HIV, transmitted drug resistance, and the paradox of preexposure prophylaxis. Proceedings of The National Academy of Sciences of The United States of America 107: 12381–12386. 20616092 10.1073/pnas.1006061107 PMC2901470

[22] Pretorius C, Stover J, Bollinger L, Bacaer N, Williams B (2010) Evaluating the Cost-Effectiveness of Pre-Exposure Prophylaxis (PrEP) and Its Impact on HIV-1 Transmission in South Africa. PLoS ONE 5: e13646. 21079767 10.1371/journal.pone.0013646 PMC2974638

[23] Dimitrov DT, Masse B, Boily MC (2010) Who Will Benefit from a Wide-Scale Introduction of Vaginal Microbicides in Developing Countries? Statistical Communications in Infectious Diseases 2(1) : Article 4. 10.2202/1948-4690.1012 PMC3905618 24490001

[24] Abbas UL, Hood G, Wetzel AW, Mellors JW (2011) Factors Influencing the Emergence and Spread of HIV Drug Resistance Arising from Rollout of Antiretroviral Pre-Exposure Prophylaxis (PrEP). PloS ONE 6: e18165. 21525976 10.1371/journal.pone.0018165 PMC3078109

[25] Dimitrov DT, Boily MC, Baggaley R, Mâsse B (2011) Modeling the Gender-Specific Impact of Vaginal Microbicides on HIV Transmission. Journal of Theoretical Biology 288: 9–20. 21840324 10.1016/j.jtbi.2011.08.001 PMC3184649

[26] Supervie V, Barrett M, Kahn JS, Musuka G, Moeti TL, et al (2011) Modeling dynamic interactions between pre-exposure prophylaxis interventions & treatment programs: predicting HIV transmission & resistance. Sci. Rep. 1: 185. 22355700 10.1038/srep00185 PMC3240958

[27] Boily MC, Dimitrov D, Karim Salim SA, Masse B (2011) The future role of rectal and vaginal microbicides to prevent HIV infection in heterosexual populations: implications for product development and prevention. SEXUALLY TRANSMITTED INFECTIONS 87(7): 646–653. 22110117 10.1136/sextrans-2011-050184 PMC3332062

[28] Hallett TB, Baeten JM, Heffron R, Barnabas R, de Bruyn G, et al (2011) Optimal Uses of Antiretrovirals for Prevention in HIV-1 Serodiscordant Heterosexual Couples in South Africa: A Modelling Study. Plos Medicine 8: e1001123. 22110407 10.1371/journal.pmed.1001123 PMC3217021

[29] Williams BG, Karim SSA, Karim QA, Gouws E (2011) Epidemiological Impact of Tenofovir Gel on the HIV Epidemic in South Africa, Jaids-Journal of Acquired Immune Deficiency Syndromes, 58: , 207–210. 10.1097/QAI.0b013e3182253c19 PMC3175282 21654503

[30] Cox AP, Foss AM, Shafer LA, Nsubuga RN, Vickerman P, et al. (2011) Attaining realistic and substantial reductions in HIV incidence: model projections of combining microbicide and male circumcision interventions in rural Uganda. Sexually Transmitted Infections, 87: , 629–634. 10.1136/sti.2010.046227 21768615

[31] Baggaley R, Powers K, Boily M (2011) What do mathematical models tell us about the emergence and spread of drug-resistant HIV? Current Opinion in HIV and AIDS 6: 131–140. 21505388 10.1097/COH.0b013e328343ad03 PMC3096989

[32] The HIV Modelling Consortium (2011) The Potential for the Spread of Drug Resistance Due to PrEP. Meeting Report. Available: http://sites.google.com/site/hivmodelling/work-packages/drug-resistance-caused-by-prep. Accessed 04 January 2013.

[33] Barditch-Crovo P, Deeks SG, Collier A, Safrin S, Coakley DF, et al (2001) Phase I/II trial of the pharmacokinetics, safety, and antiretroviral activity of tenofovir disoproxil fumarate in human immunodeficiency virus-infected adults. Antimicrobial Agents and Chemotherapy 45: 2733–2739. 11557462 10.1128/AAC.45.10.2733-2739.2001 PMC90724

[34] Louie M, Hogan C, Hurley A, Simon V, Chung C, et al (2003) Determining the antiviral activity of tenofovir disoproxil fumarate in treatment-naive chronically HIV-1-infected individuals. AIDS 17: 1151–1156. 12819516 10.1097/00002030-200305230-00006

[35] Prada N, Davis B, Jean-Pierre P, La Roche M, Duh FM, et al (2008) Drug-susceptible HIV-1 infection despite intermittent fixed-dose combination tenofovir/emtricitabine as prophylaxis is associated with low-level viremia, delayed seroconversion, and an attenuated clinical course. Jaids 49: 117–122. 18769360 10.1097/QAI.0b013e3181869a9b PMC2689390

[36] Statistics South Africa (2010) Mid-year population estimates. Available: http://www.statssa.gov.za/publications/P0302/P03022010.pdf. Accessed 04 Jan 2013.

[37] Rehle TM, Hallett TB, Shisana O, Pillay-van Wyk V, Zuma K, et al (2010) A Decline in New HIV Infections in South Africa: Estimating HIV Incidence from Three National HIV Surveys in 2002, 2005 and 2008. Plos One 5: e11094. 20559425 10.1371/journal.pone.0011094 PMC2885415

[38] Boily MC, Baggaley RF, Wang L, Masse B, White RG, et al (2009) Heterosexual risk of HIV-1 infection per sexual act: systematic review and meta-analysis of observational studies. Lancet Infectious Diseases 9: 118–129. 19179227 10.1016/S1473-3099(09)70021-0 PMC4467783

[39] UNAIDS/WHO (2009) AIDS epidemic update: November 2009 UNAIDS/World Health Organization, Geneva. Available: http://www.unaids.org/en/media/unaids/contentassets/dataimport/pub/report/2009/jc1700_epi_update_2009_en.pdf. Accessed 13 Sept 2013.

[40] Ferry B, Carael M, Buve A, Auvert B, Laourou M, et al (2001) Comparison of key parameters of sexual behaviour in four African urban populations with different levels of HIV infection. AIDS 15: S41–S50. 10.1097/00002030-200108004-00005 11686464

[41] Morgan D, Mahe C, Mayanja B, Okongo JM, Lubega R, et al (2002) HIV-1 infection in rural Africa: is there a difference in median time to AIDS and survival compared with that in industrialized countries? AIDS 16: 597–603. 11873003 10.1097/00002030-200203080-00011

[42] Porter K, Zaba B (2004) The empirical evidence for the impact of HIV on adult mortality in the developing world: data from serological studies. AIDS 18: S9–S17. 10.1097/00002030-200406002-00002 15319739

[43] Johnson L, Dorrington R, Bradshaw D, Pillay-Van Wyk V, Rehle T (2009) Sexual behaviour patterns in South Africa and their association with the spread of HIV: insights from a mathematical model. Demographic Research 21: 289–340.

[44] Kalichman SC, Simbayi LC, Cain D, Jooste S (2009) Heterosexual anal intercourse among community and clinical settings in Cape Town, South Africa. Sexually Transmitted Infections 85: 411–415. 19429569 10.1136/sti.2008.035287 PMC3017216

[45] Todd J, Cremin I, McGrath N, Bwanika J-B, Wringe A, et al.. (2009) Reported number of sexual partners: comparison of data from four African longitudinal studies Sexually Transmitted Infections, 85, i72–i80 10.1136/sti.2008.033985 PMC2654146 19307344

[46] Foss AM, Hossain M, Vickerman PT, Watts CH (2007) A systematic review of published evidence on intervention impact on condom use in sub-Saharan Africa and Asia. Sexually Transmitted Infections 83: 510–516. 17932124 10.1136/sti.2007.027144 PMC2598651

