# Supplementary material for: Analytic Review of Modeling Studies of ARV Based PrEP Interventions Reveals Strong Influence of Drug-Resistance Assumptions on the Population-Level Effectiveness
Source: PLoS One. 2013 Nov 25;8(11):e80927. doi: 10.1371/journal.pone.0080927 (PMC3840039; doi:10.1371/journal.pone.0080927)
Supplement: File S1 — Supporting Information (DOCX) [file pone.0080927.s001.docx]

**Supporting Information**

**Analytic review of modeling studies of ARV based PrEP interventions reveals strong influence of drug-resistance assumptions on the population-level effectiveness**

**Dobromir Dimitrov, Marie-Claude Boily, Elizabeth R. Brown, Tim Hallett**

**1. Description and structure of the mathematical model**

Our model (Figure 1 in the main text) is implemented through a system of differential equations which govern the flows between the following population compartments:

$S_{g}^{p}$ - susceptible women (g=w) and men (g=m) using PrEP

$S_{g}$ - susceptible women (g=w) and men (g=m) not using PrEP

$I_{g}^{p}$- HIV-positive women (g=w) and men (g=m) using PrEP infected with the wild type HIV

$I_{g}$ - HIV-positive women (g=w) and men (g=m) not using PrEP infected with the wild type HIV

$I_{rg}$, $I_{rg}^{p}$ - HIV-positive women (g=w) and men (g=m) not using or using PrEP who developed drug-resistance (acquired resistance, ADR)

$I_{Rg},I_{Rg}^{p}$ - HIV-positive women (g=w) and men (g=m) not using or using PrEP who were infected with drug-resistant HIV (transmitted resistance, TDR)

$A_{g}$- women (g=w) and men (g=m) who developed AIDS or died from AIDS

Model equations which describe the rates of change in the population compartments corresponding to one gender are:

$$\begin{matrix} \frac{dS_{g}}{dt}=(1-k)\Lambda_{g}-(\lambda_{g}+\lambda_{rg})S_{g}-\mu S_{g} \\ \frac{dS_{g}^{p}}{dt}=k\Lambda_{g}-(\lambda_{g}^{p}+\lambda_{rg}^{p})S_{g}^{p}-\mu S_{g}^{p} \\ \frac{dI_{g}}{dt}=\lambda_{g}S_{g}+\delta I_{g}^{p}+\tau I_{rg}+\tau_{1} I_{Rg}-(\mu+d )I_{g} \\ \frac{dI_{Rg}}{dt}=\lambda_{rg}S_{g}+\delta I_{Rg}^{p}-\tau_{1} I_{Rg}-(\mu+d_{r}) I_{Rg} \\ \frac{dI_{rg}}{dt}=\delta I_{rg}^{p}-\tau I_{rg}-(\mu+d_{r}) I_{rg} \\ \frac{dI_{g}^{p}}{dt}=\lambda_{g}^{p}S_{g}^{p}-(r+\delta+\mu+d) I_{g}^{p} \\ \frac{dI_{Rg}^{p}}{dt}=\lambda_{rg}^{p}S_{g}^{p}-(\delta+\mu+d_{r}) I_{Rg}^{p} \\ \frac{dI_{rg}^{p}}{dt}=rI_{g}^{p}-(\delta+\mu+d_{r}) I_{rg}^{p} \\ \frac{dA_{g}}{dt}=d(I_{g}+I_{g}^{p})+d_{r}(I_{rg}+I_{rg}^{p}+I_{Rg}+I_{Rg}^{p}) \end{matrix}$$

The recruitment rate *Λ_g_* in each gender is selected to preserve the size of a HIV-free population, i.e., *Λ_g_* =*μN_g_* where $N_{g}=S_{g}+S_{g}^{p}+I_{g}+I_{g}^{p}+I_{rg}+I_{rg}^{p}+I_{Rg}+I_{Rg}^{p}$ - represent the sexually active males (g=m) or females (g=w), respectively.

PrEP is prescribed to a proportion *k* of the HIV-negative of the individuals entering the population. The biological meaning of the parameters and their ranges are given in Table 1.

**2. Forces of infection**

The forces of infections ($\lambda_{g},\lambda_{rg},\lambda_{g}^{p}, and \lambda_{rg}^{p}$) in gender ($g$) are based on number of sex partners per year (*ρ_g_*), annual acquisition risk ($R_{g}^{i,j}$and $R_{rg}^{i,j}$ *)* for uninfected individual not using PrEP (i=0) or using PrEP (i=p) per partnership with infected individual not using PrEP (j=0), or using PrEP (j=p) and the fraction of the opposite gender ($\bar{g}$) which is currently in the specific compartment. The subscript r indicates that the partner is infected with drug-resistant HIV:

The probability to acquired resistant virus in a contact with resistance carrier is $\varepsilon$ if the partner has developed resistance when on PrEP (ADR) or $\varepsilon_{1}$if the partner has been infected with resistant HIV (TDR).

Annual risks per partnership ($R_{g}^{i,j}$and $R_{rg}^{i,j}$ *)* are derived from standard binomial models based on the number of sex acts per partnership (*n_g_*/ρ_g_), the fraction of sex acts protected by condom (c) and the HIV acquisition risk per sex act which depends on which partners are using PrEP and on the partners’ resistance status:

$$\begin{matrix} R_{g}^{0,0}=1-(1-(1-\alpha_{c})\beta_{g})^{cn_{g}/\rho{}_{g}}(1-\beta_{g})^{(1-c)n_{g}/\rho_{g}} \\ R_{rg}^{0,0}=1-(1-(1-\alpha_{c})\beta_{r}\beta_{g})^{cn_{g}/\rho_{g}}(1-\beta_{r}\beta_{g})^{(1-c)n_{g}/\rho_{g}} \\ R_{g}^{p,0}=1-(1-(1-\alpha_{c})(1-\alpha_{s})\beta_{g})^{cn_{g}/\rho_{g}}(1-(1-\alpha_{s})\beta_{g})^{(1-c)n_{g}/\rho_{g}} \\ R_{rg}^{p,0}=1-(1-(1-\alpha_{c})(1-\gamma_{r}\alpha_{s})\beta_{r}\beta_{g})^{cn_{g}/\rho_{g}}(1-(1-\gamma_{r}\alpha_{s})\beta_{r}\beta_{g})^{(1-c)n_{g}/\rho_{g}} \\ R_{g}^{0,p}=1-(1-(1-\alpha_{c})(1-\alpha_{i})\beta_{g})^{cn_{g}/\rho_{g}}(1-(1-\alpha_{i})\beta_{g})^{(1-c)n_{g}/\rho_{g}} \\ R_{rg}^{0,p}=1-(1-(1-\alpha_{c})(1-\gamma_{r}\alpha_{i})\beta_{r}\beta_{g})^{cn_{g}/\rho_{g}}(1-(1-\gamma_{r}\alpha_{i})\beta_{r}\beta_{g})^{(1-c)n_{g}/\rho_{g}} \\ R_{g}^{p,p}=1-(1-(1-\alpha_{c})(1-\alpha_{i})(1-\alpha_{s})\beta_{g})^{cn_{g}/\rho_{g}}(1-(1-\alpha_{i})(1-\alpha_{s})\beta_{g})^{(1-c)n_{g}/\rho_{g}} \\ R_{rg}^{p,p}=1-(1-(1-\alpha_{c})(1-\gamma_{r}\alpha_{i})(1-\gamma_{r}\alpha_{s})\beta_{r}\beta_{g})^{cn_{g}/\rho_{g}}(1-(1-\gamma_{r}\alpha_{i})(1-\gamma_{r}\alpha_{s})\beta_{r}\beta_{g})^{(1-c)n_{g}/\rho_{g}} \end{matrix}$$

Here β_g_ is the female (g=w) and male (g=m) HIV acquisition risk per unprotected vaginal act if neither partner uses PrEP and the infected partner carry wild type HIV, β_r_ is the relative infectiousness of individuals infected with drug-resistant HIV compared to in infected with wild-type, α_s_ (α_i_) measures the efficacy of PrEP in reducing susceptibility (infectiousness) of PrEP users, while γ_r_ is the relative PrEP efficacy when exposed to drug-resistant compared to wild-type HIV.

**3. Epidemic conditions at the start of PrEP intervention**

PrEP interventions are initiated in populations with equal representation of the sexes (*N_m_*(0)=*N_w_*(0)) with predefined HIV prevalence in women (*P_w_)* and men (*P_m_)*. PrEP is initially prescribed to a proportion *k_1_* of the HIV-negative and to a reduced proportion of (1-θ) *k_1_* of the HIV-positive individuals. Based on that the initial size of the population compartments is set as follows:

$$\begin{matrix} S_{g}(0)=(1-k_{1})(1-P_{g})N_{g}(0) \\ S_{g}^{p}(0)=k_{1}(1-P_{g})N_{g}(0) \\ I_{g}(0)=(1-(1-\theta)k_{1})P_{g}N_{g}(0) \\ I_{g}^{p}(0)=(1-\theta)k_{1}PN_{g}(0) \\ I_{rg}^{p}(0)=I_{Rg}^{p}(0)=0 \\ I_{rg}(0)=I_{Rg}(0)=0 \end{matrix}$$

**4. Additional results**


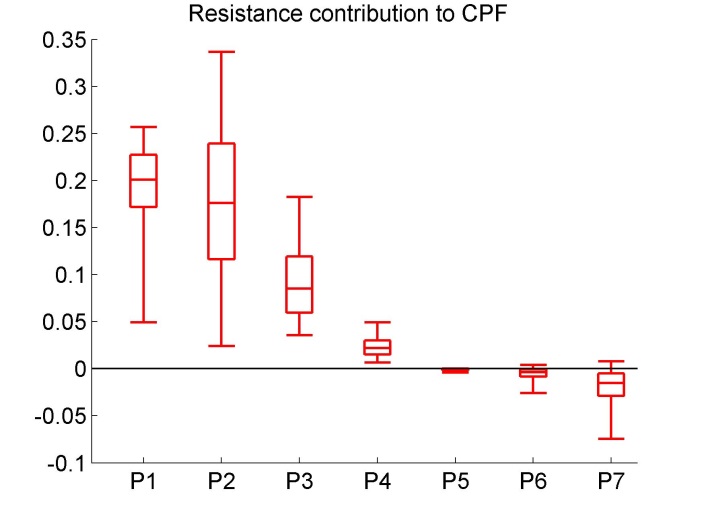

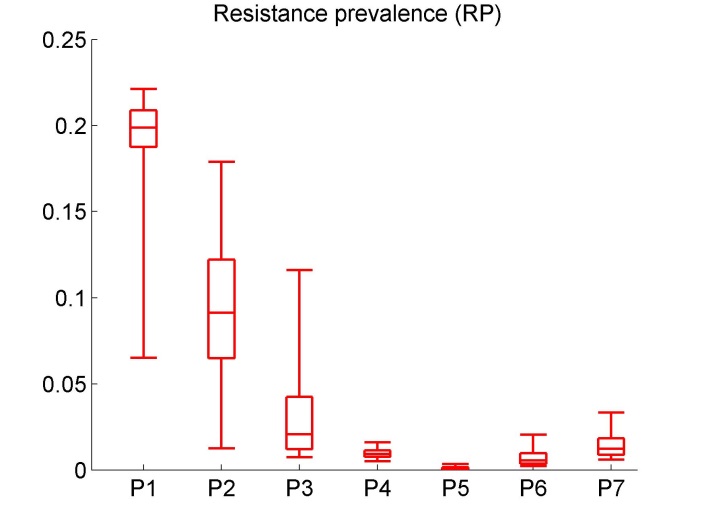

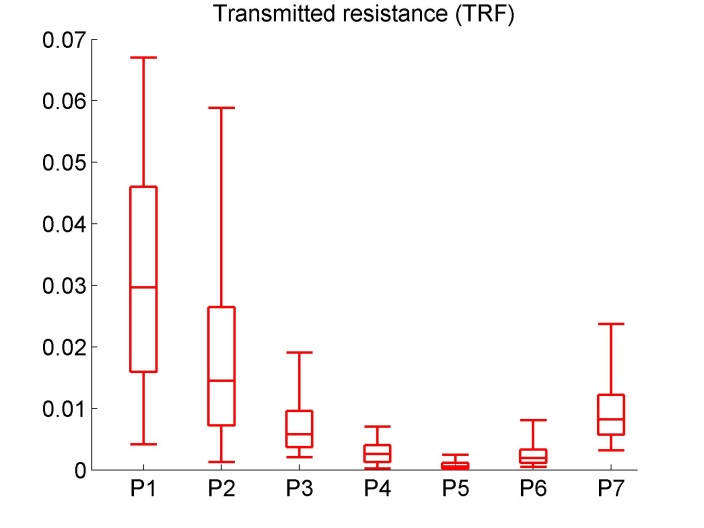

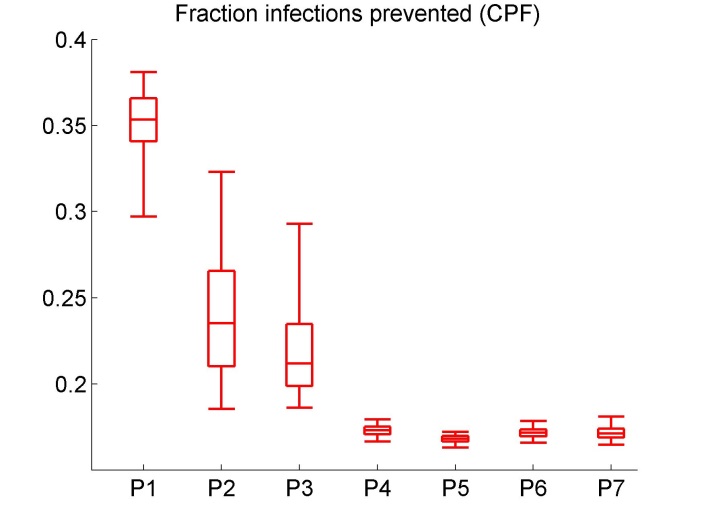


A)

B)

C)

D)

Figure S1 Projected impact of 10 years of PrEP use under the scenarios with 25% coverage on A) the cumulative fraction of prevented infections (CPF); B) resistance prevalence due to PrEP (RP); C) cumulative fraction of infections in which resistance is transmitted (TRF) and D) resistance contribution to CPF when the model is parameterized based on the resistance assumptions extracted from published papers. The boxplots (median, 2.5th, 25th, 75th, 97.5th percentiles) reflect the variation in impact estimates based on 10,000 simulations (10 per preselected epidemic set). In D, the contribution of resistance to CPF is calculated as the percentage change in CPF from simulations in which the resistance is disregarded. Intervention parameters are fixed on their baseline values from Table 1, part C.


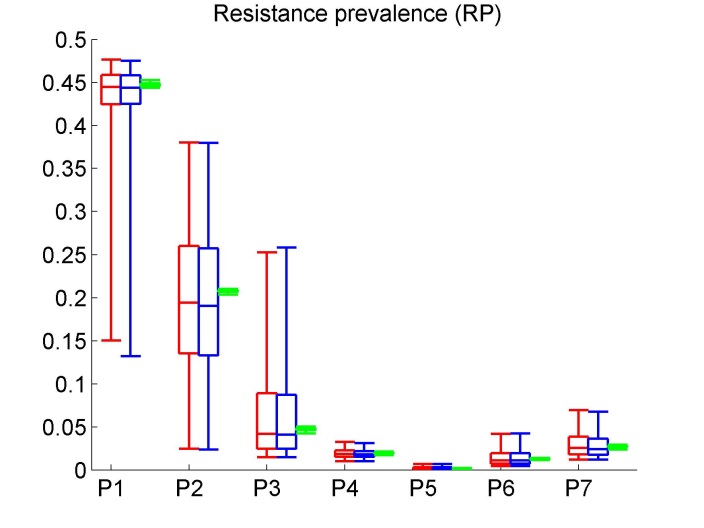

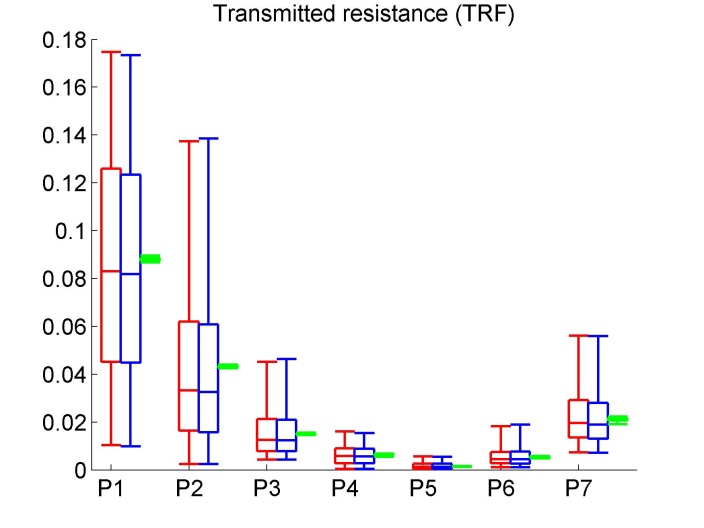

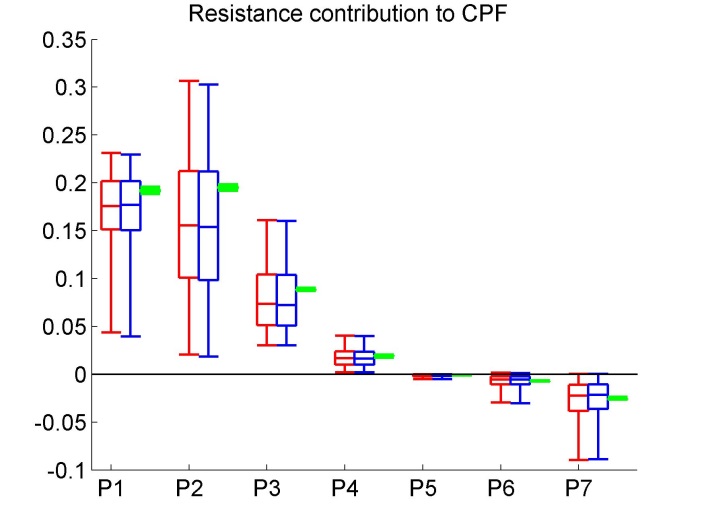

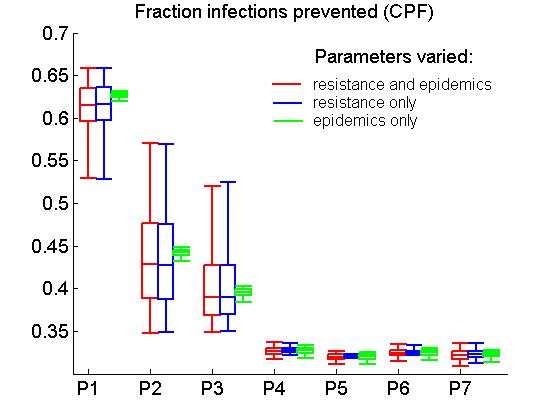


A)

B)

C)

D)

Figure S2 Projected impact of 10 years of PrEP use on A) the cumulative fraction of prevented infections (CPF); B) resistance prevalence due to PrEP (RP); C) cumulative fraction of infections in which resistance is transmitted (TRF) and D) resistance contribution to CPF when resistance parameters and epidemic parameters are varied (red), only resistance parameters are varied (blue) or only epidemic parameters are varied (green). The boxplots (median, 2.5th, 25th, 75th, 97.5th percentiles) reflect the variation in impact estimates based on 10,000 simulations. In D), the contribution of resistance to CPF is calculated as the percentage change in CPF from simulations in which the resistance is disregarded. Intervention parameters are fixed on their baseline values from Table 1, part C.


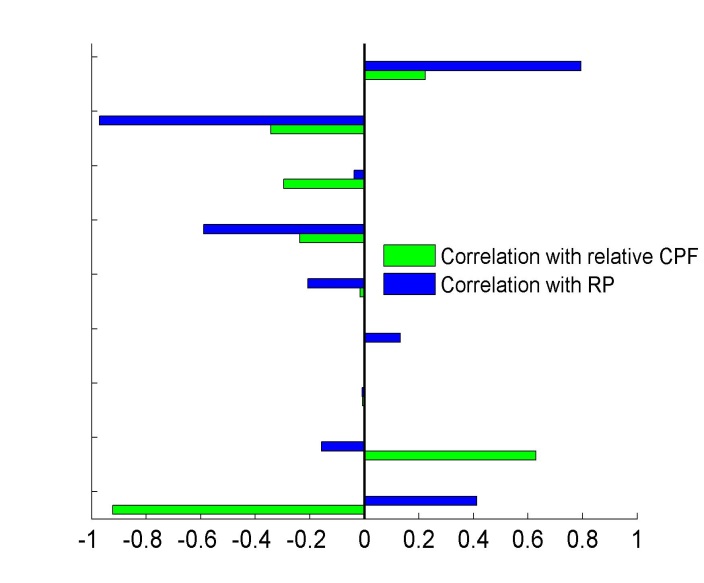


Resistance parameters:

| Resistance rate, full adherers |
| --- |
| PrEP drop rate, HIV+ users |
| Reduction in the initial fraction of HIV+ PrEP users |
| Reversion of resistance acquired on PrEP |
| Reversion of transmitted resistance |
| Resistance transmission from partner with ADR |
| Resistance transmission from partner with TDR |
| Relative infectiousness of resistance carriers |
| Relative PrEP eff. when exposed to resistant HIV |

**Figure S3** Partial rank correlation coefficients (PRCC) between parameters which affect resistance and intervention outcomes, relative 10-year CPF (green) and resistance prevalence after 10 years (blue) based on 10, 000 simulations with fixed epidemic conditions. Intervention parameters are also fixed on their baseline values from Table 1, part C. Relative CPF is calculated as the ratio of the 10-year CPF for scenarios with resistance over baseline scenario (no resistance).

**
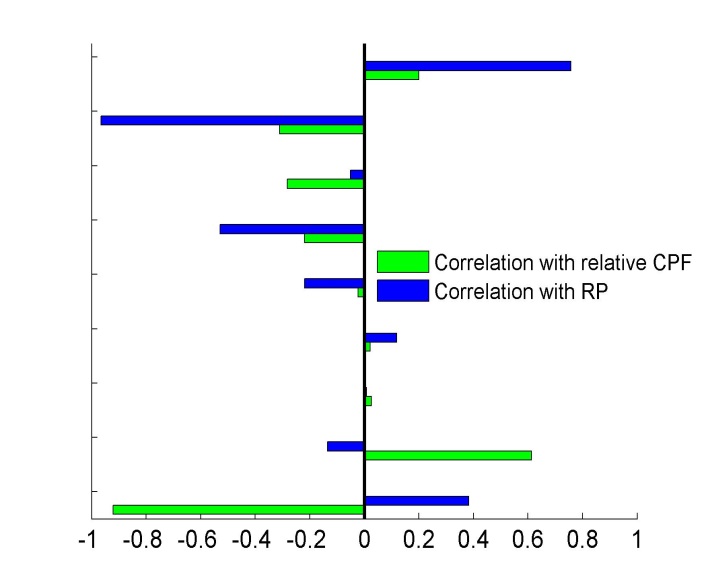
**
